# [^18^F]tetrafluoroborate as a PET tracer for the sodium/iodide symporter: the importance of specific activity

**DOI:** 10.1186/s13550-016-0188-5

**Published:** 2016-04-22

**Authors:** Alex Khoshnevisan, Maite Jauregui-Osoro, Karen Shaw, Julia Baguña Torres, Jennifer D. Young, Nisha K. Ramakrishnan, Alex Jackson, Gareth E. Smith, Antony D. Gee, Philip J. Blower

**Affiliations:** Division of Imaging Sciences and Biomedical Engineering, King’s College London, 4th Floor Lambeth Wing, St. Thomas’ Hospital, London, SE1 7EH UK; The Grove Centre, GE Healthcare, White Lion Road, Amersham, UK

**Keywords:** Sodium/iodide symporter, Tetrafluoroborate, Fluorine-18, PET, Specific activity, Thyroid

## Abstract

**Background:**

[^18^F]BF_4_^−^, the first ^18^F-labelled PET imaging agent for the sodium/iodide symporter (NIS), was produced by isotopic exchange yielding a product with limited specific activity (SA, ca. 1 GBq/μmol) posing a risk of sub-optimal target-to-background ratios (TBR) in PET images due to saturation of NIS in vivo. We sought to quantify this risk and to develop a method of production of [^18^F]BF_4_^−^ with higher SA.

**Methods:**

A new radiosynthesis of [^18^F]BF_4_^−^ was developed, involving reaction of [^18^F]F^−^ with boron trifluoride diethyl etherate under anhydrous conditions, guided by ^11^B and ^19^F NMR studies of equilibria involving BF_4_^−^ and BF_3_. The SA of the product was determined by ion chromatography. The IC_50_ of [^19^F]BF_4_^−^ as an inhibitor of [^18^F]BF_4_^−^ uptake was determined in vitro using HCT116-C19 human colon cancer cells expressing the human form of NIS (hNIS). The influence of [^19^F]BF_4_^−^ dose on biodistribution in vivo was evaluated in normal mice by nanoPET imaging and ex vivo tissue counting.

**Results:**

An IC_50_ of 4.8 μΜ was found in vitro indicating a significant risk of in vivo NIS saturation at SA achieved by the isotopic exchange labelling method. In vivo thyroid and salivary gland uptake decreased significantly with [^19^F]BF_4_^−^ doses above ca. 10 μg/kg. The new radiosynthesis gave high radiochemical purity (>99 %) and moderate yield (15 %) and improved SA (>5 GBq/μmol) from a starting activity of only 1.5 GBq.

**Conclusions:**

[^18^F]BF_4_^−^ produced at previously reported levels of SA (1 GBq/μmol) can lead to reduced uptake in NIS-expressing tissues in mice. This is much less likely in humans. The synthetic approach described provides an alternative for production of [^18^F]BF_4_^−^ at higher SA with sufficient yield and without need for unusually high starting activity of [^18^F]fluoride, removing the risk of NIS saturation in vivo even in mice.

**Trial registration:**

ISRCTN75827286.

**Electronic supplementary material:**

The online version of this article (doi:10.1186/s13550-016-0188-5) contains supplementary material, which is available to authorized users.

## Background

The Na^+^/I^−^ symporter (NIS) is an important molecular target in the field of nuclear medicine [1, 2]. Accumulation of radioiodide at sites of NIS expression such as salivary gland, gastric mucosa and lactating mammary gland [3] and especially thyroid and thyroid cancers [4], has found use in gamma camera imaging using iodide in the form of [^131^I] and [^123^I]I^−^ and radionuclide therapy using [^131^I]I^−^. In addition, following the cloning of its human form human sodium/iodide symporter (hNIS) [5], it has been widely used in reporter gene imaging to study in vivo trafficking of metastatic cancer cells [6], stem cells [7] and immune cells [8]. Anion transport by NIS is not specific to iodide [9]; other anions with similar charge and ionic radius are also transported (Additional file 1) including [^99m^Tc]pertechnetate, which is an important NIS tracer for single photon emission computed tomography (SPECT) imaging, and [^186^Re]perrhenate [10], [^188^Re]perrhenate [11] and [^211^At]astatide [12, 13], which have potential for NIS-targeted radionuclide therapy. While planar scintigraphy and SPECT have been the mainstay of clinical molecular imaging of hNIS, a positron emission tomography (PET) imaging agent would potentially bring improved sensitivity, resolution and quantification. PET imaging with [^124^I]I^−^ has been shown to enhance sensitivity [14] compared to imaging with [^131^I]I^−^, but its long half-life, high positron energy and low positron yield are problematic for both dosimetry and image quality. The potential of [^18^F]BF_4_^−^ as a PET tracer for imaging NIS was recently reported in mice [15] and non-human primates [16]. This offered improved image quality and dosimetry compared to SPECT and ^124^I PET, as well as in vitro and in vivo stability and ease of production in PET centres. The radiolabelling method for [^18^F]BF_4_^−^ entailed isotopic exchange of [^18^F]F^−^ with [^19^F]BF_4_^−^ under acidic aqueous conditions [15] leading to a final product with a relatively low specific activity (SA) of 1 GBq/μmol (i.e. with only one in 6.25 × 10^6^ BF_4_^−^ ions labelled), from a starting activity of 10 GBq. With a reported [17] IC_50_ of 1.6 μM for [^19^F]BF_4_^−^ in vitro, this low SA presents a risk that NIS saturation may occur in vivo, reducing uptake in target tissues when activities sufficient for high-quality PET imaging are injected, especially in mice [18] owing to higher injected radioactivity per kilogram.

In this investigation, we sought to quantify the minimum SA required to combine good-quality PET images in mice with maximum target-to-background ratio (TBR) in NIS-expressing tissue and to develop an alternative method of radiolabelling that gives sufficient SA to avoid the risk of saturation and sub-optimal TBR. A radiolabelling strategy that does not entail isotopic exchange would be expected to lead to higher SA. Here, we report production of [^18^F]BF_4_^−^ predicated on addition of [^18^F]fluoride to BF_3_ rather than isotopic exchange with [^19^F]BF_4_^−^.

## Methods

### General

Reagents and materials were purchased from Sigma-Aldrich (Gillingham, UK) unless otherwise stated. Ultrapure water (resistivity > 18.2 MΩ) was used throughout. [^18^F]F^−^ was obtained by proton irradiation of [^18^O]H_2_O (97 at.%, Rotem Industries Ltd., Israel) with a CTI RDS 112 cyclotron (11 MeV, 30 μA beam current). Quaternary methyl ammonium (QMA) cartridges (Sep-Pak QMA Light, Waters, UK) were preconditioned with 1 M NaCl (5 mL) and H_2_O (10 mL), and neutral alumina cartridges (Alumina N Plus Lite Sep-Pak, Waters, UK) were preconditioned with H_2_O (10 mL), acetone (10 mL) and air (10 mL) unless otherwise stated.

### NMR

In order to determine the concentration of 15-crown-5 (15C5) in formulated [^18^F]BF_4_^−^, ^1^H spectra were acquired of decayed [^18^F]BF_4_^−^ samples in the presence of an internal reference using a Bruker Ultrashield 400WB PLUS 9.4-T spectrometer. The 15C5 integral with respect to that of the internal reference was then compared to that of 15C5 standards (5–0.01 mg/mL) of known concentration. Both standards and samples were analysed as solutions in D_2_O/H_2_O (3:2) with potassium hydrogen phthalate (1 mg/mL) as the internal reference. Data were analysed using MestReNova LITE (v5.2.5). ^19^F and ^11^B nuclear magnetic resonance (NMR) spectra were acquired using a Bruker AVIII Ultrashield Plus WB with a field strength of 9.4 T, operating at frequencies of 375.878 and 128.166 MHz for ^19^F and ^11^B, respectively. Data were acquired and processed using Topspin 2.1.

Hydrolysis of NaBF_4_ was studied by ^19^F and ^11^B NMR in water and under conditions similar to those used for the isotopic exchange synthesis of [^18^F]BF_4_^−^ [15] but without ^18^F. Briefly, NaBF_4_ was dissolved in 1.0 M hydrochloric acid at a concentration of 4 mg/mL. The solution (5 mL) was heated to 100 °C for 10 min, cooled to 25 °C and passed through a silver ion-loaded cation exchange cartridge (OnGuard II AG, Dionex, Leeds, UK, conditioned with 10 mL water and 10 mL air) to remove chloride and raise the pH, and then through an alumina column (Alumina N Plus Lite Sep-Pak, Waters, UK, conditioned with 10 mL water and 5 mL air) and a sterile Millex-GS 0.22-μm filter unit (Millipore UK, Watford, UK). ^19^F and ^11^B NMR spectra of the sample were acquired at each stage of the process.

### Radiochemistry optimisation

A range of conditions were examined for the reaction of [^18^F]F^−^ with boron trifluoride diethyl etherate (BF_3_·OEt_2_). Briefly, [^18^F]F^-^ was trapped by passing the irradiated [^18^O]H_2_O through a QMA cartridge and eluted with various salt solutions depending on which fluoride salt was required. The [^18^F]F^−^ solution was then dried manually in a glass vial by repeated azeotropic distillation with acetonitrile (MeCN) (3 × 0.5 mL), before adding BF_3_·OEt_2_ (10–0.0001 μL) in MeCN (1 mL, solutions prepared by serial dilution). The reaction was then allowed to occur, with or without elevated temperature, before quenching with H_2_O (1 mL) and analysing by radioTLC to determine crude radiochemical yield (RCY) by radioTLC (see below) as a measure of reaction step efficiency. Further details of conditions and results are shown in Additional file 2.

### Radioanalytical methods

RadioTLC was carried out using a neutral alumina stationary phase (Macherey-Nagel, 10 × 80 mm, Polygram ALOX N/UV_254_) with methanol (100 %) as the mobile phase. The thin-layer chromatography (TLC) plates were scanned using a radioTLC linear scanner (LabLogic Mini-Scan™) with β^+^ probe (LabLogic B-FC-3600). The purity of the crude product in the reaction solution was determined as the radioactivity associated with the BF_4_^−^ peak (*R*_f_ = 0.6, c.f. *R*_f_ = 0 for fluoride) as a percentage of the total detected chromatogram radioactivity. Radiochemical identity and purity of the final product were measured by ion chromatography (Metrohm 930 Compact IC Flex) with in-line conductimetric and gamma detectors using a Shodex IC I-524A column (4.6 × 100 mm) with 2.3 mM phthalic acid and 2.3 mM tris(hydroxymethyl)aminomethane (pH 5.0) in H_2_O as the eluent. The flow rate was 1.5 mL/min, and column temperature was 40 °C. The concentration of [^19/18^F]NaBF_4_ in the final product was determined from the ion chromatography data by reference to a calibration curve.

### Optimised radiosynthesis and automation

[^18^F]F^−^ in H_2_O was trapped on a QMA ion exchange column and eluted with 0.9 % NaCl (0.5 mL). The eluate was then dried under a stream of N_2_ at 95 °C, followed by azeotropic distillation with MeCN (3 × 0.5 mL). 15C5 (24 mg) in MeCN (0.5 mL) and BF_3_·OEt_2_ (0.1 μL (0.8 μM), prepared by serial dilution) in MeCN (0.5 mL) were then added and the mixture heated to 80 °C for 10 min. The reaction mixture was passed over a neutral alumina cartridge into a vial containing H_2_O (1 mL). This mixture was then passed over a QMA cartridge. The alumina and QMA cartridges in tandem were washed with H_2_O (2 mL), and the QMA cartridge containing the product was then washed with further H_2_O (4 mL). The purified product was then eluted from the QMA cartridge with 0.9 % NaCl (0.5 mL). This protocol was automated using a GE FASTLab™ with a custom cassette layout and ~1.5-GBq starting radioactivity (see Additional file 3).

### Cellular uptake study

HCT116-hNIS-C19 cells (hNIS-transfected human colon carcinoma cell line [17]) were seeded in 12-well plates at a density of 0.5 × 10^6^ cells/well and incubated with 5 % CO_2_ at 37 °C for 24 h prior to experiments. Each well was washed twice with Hanks’ balanced salt solution (HBSS) before incubation with [^19^F]NaBF_4_ in HBSS (700 μL) for 30 min. [^18^F]NaBF_4_ (0.1 MBq, produced by method described above) in HBSS (50 μL) was then added to give a final concentration of [^19^F]BF_4_^−^ ranging from 10^−2^ to 10^−12^ M. The plates were then incubated at 37 °C for a further 30 min. The medium was then removed from each well and the cells washed with HBSS (2 × 750 μL) and the medium and washings reserved for counting. Cell-bound activity was then extracted with 1 M NaOH (750 μL). Bound and unbound radioactivity were then measured in a gamma counter and the uptake of the radiotracer expressed as a percentage of the total radioactivity per well.

### PET imaging

Imaging experiments were performed using a nanoScan® PET/CT (Mediso Medical Imaging Systems, Budapest, Hungary). PET/computed tomography (CT) imaging of [^18^F]BF_4_^−^ produced by the optimised high-specific-activity methods described above was performed in 4–8-week-old female BALB/c mice (*n* = 3 per SA group). Animals were anaesthetised by isoflurane inhalation (3 %, Animalcare, York, UK, in oxygen) and placed on the scan bed in a prone position. The SA of the [^18^F]BF_4_^−^ radiotracer was adjusted to produce five samples with SA of 5, 1, 0.2, 0.1 and 0.01 GBq/μmol by addition of [^19^F]NaBF_4_ in 0.9 % NaCl. With an injected radioactivity of 2.5 MBq (≤150 μL) per mouse, these correspond to injected BF_4_^−^ doses of 25, 125, 625, 1250 and 12,500 nmol/kg. Syringe activity was measured before and after injection using a dose calibrator to determine injected activity. Each sample with chosen SA was injected into one of five groups of mice (*n* = 3, 2.5 MBq per mouse) through a cannula inserted into the lateral tail vein. A sixth group was injected with only 0.5 MBq of the 5 GBq/μmol sample to achieve a reduced total BF_4_^−^ dose of 5 nmol/kg. Dynamic PET (400–600-keV energy window; 1:5 coincidence mode; 5-ns coincidence window, 0.30 × 0.30 × 0.30 mm^3^ voxel size) was acquired for 30 min followed by a CT scan (180 projections, 45 kVp, 0.25 × 0.25 × 0.21 mm^3^ voxel size). Respiration rate and bed temperature were monitored throughout the scan. Anaesthesia was maintained at 2–2.5 % isoflurane during scanning.

### PET image analysis

All PET/CT datasets were reconstructed using the Monte Carlo-based full-3D iterative algorithm Tera-Tomo (Mediso Medical Imaging Systems, Budapest, Hungary) [19]. Raw PET data were reconstructed into 5-min bins using reconstruction settings (4 iterations, 6 subsets, 0.4 × 0.4 × 0.4 mm^3^ voxel size) as well as intercrystal scatter correction. Decay correction to injection time was applied. All reconstructed datasets were analysed using VivoQuant software (v2.0, inviCRO, LLC, Boston, USA). Regions of interest (ROIs) for different organs (thyroid, salivary glands, stomach and bladder) were manually defined around each organ (*vide infra*) in order to express ^18^F uptake in each organ as a standardised uptake value (SUV). The SUV was calculated as the ratio of radioactivity in each ROI (MBq) per gram of organ tissue (weighed post-mortem) and radioactivity in the whole-body ROI (MBq) per whole-body weight (excluding tail). The total radioactivity present in each organ was determined by drawing an approximate ROI encompassing the whole organ, using a threshold of 10 % of the maximum count to define the edge of the ROI. The binned images corresponding to 25–30 min post-injection were used for the ROI analysis. For calculations involving the thyroid, which is difficult to dissociate from trachea and weigh accurately, a previously reported standard thyroid tissue mass of 3.6 mg [15, 20] was used as the weight of the organ.

### Ex vivo biodistribution

At the end of the imaging experiment, mice were culled by cervical dislocation (45 min post-injection) and all major organs were explanted, weighed and gamma-counted (LKB Wallac 1282). Urine expelled during cervical dislocation by some animals was collected on absorbent material, and this radioactivity was counted and considered as part of the urine/bladder activity. The thyroid was extracted while attached to the trachea, and a standard thyroid tissue mass of 3.6 mg was used for reasons explained above. ^18^F concentration in each organ was expressed as SUV (also presented as % ID/g in Additional files 4 and 5). The total injected dose was defined as the sum of organ counts (including excreted activity) and carcass counts (excluding tail).

## Results

### In vitro ^19^F-BF_4_^−^ competition study

**Fig. 1:**
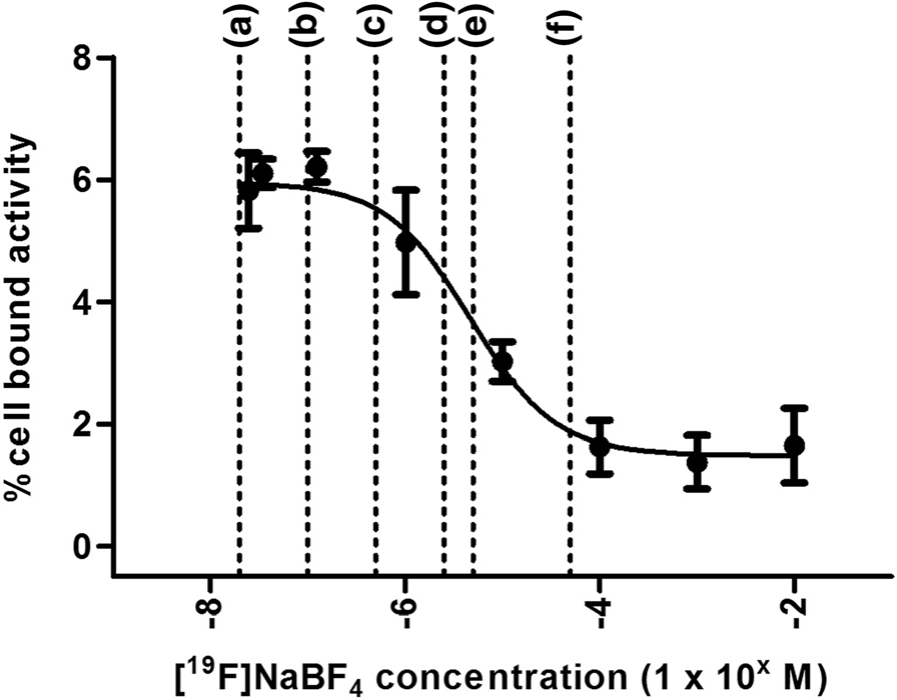
Inhibition curve for blocking [^18^F]BF_4_^−^ accumulation with ^19^F-BF_4_^−^ in vitro in HCT116-hNIS-C19 cells. *Dashed lines* labelled (*a*) to (*f*) represent estimated prospective in vivo initial extracellular concentrations of ^18/19^F-BF_4_^−^ at varying SA following injection of ~2.5 MBq radiotracer into a mouse: (*a*) 5 nmol/kg, (*b*) 25 nmol/kg, (*c*) 125 nmol/kg, (*d*) 625 nmol/kg, (*e*) 1250 nmol/kg and (*f*) 12.5 μmol/kg. *Error bars* represent 1 SD

Varying the concentration of ^19^F-BF_4_^−^ to inhibit uptake of tracer levels of [^18^F]BF_4_^−^ produced by the method described here gave a typical saturable sigmoid curve (Fig. 1) from which an IC_50_ of 4.7 μM was calculated, in reasonable agreement with previously reported values [17]. Uptake was measured at 30 min and has previously been examined over periods of up to 2 h [15, 17]. Thus, as expected, carrier BF_4_^−^ at sufficient concentrations (>0.1 μM) can reduce uptake of tracer [^18^F]NaBF_4_. Using [^18^F]BF_4_^−^ at specific activities in the range previously reported, initial in vivo extracellular BF_4_^−^ concentration may well exceed this level after administration of radioactivity doses sufficient for high-quality PET imaging in mice. Therefore, in order to assess the effect of SA, [^18^F]BF_4_^−^ samples of higher SA than those previously reported [15] were required which could then be diluted to the required SA.

### NMR study of BF_4_^−^ purification and hydrolysis equilibria

To understand the factors that limit specific activity during production, investigation of the chemical processes occurring during the published [15] aqueous production and purification of [^18^F]BF_4_^−^ were conducted using ^11^B and ^19^F NMR spectrometry. NaBF_4_ in solution (H_2_O and D_2_O) showed a single peak in the ^11^B spectrum (*δ* −0.5 ppm, quintet, *J* = 1.3 Hz, Additional file 6) and two peaks in the ^19^F (*δ* −151.52 ppm, septet, and *δ* −151.57 ppm, quartet, *J* = 0.5 and 1.3 Hz, respectively, Additional file 7) corresponding to the ratio of ^10^B/^11^B isotopic abundance (septet and quartet, respectively). After addition of 1.5 M HCl to this NaBF_4_ solution, an additional species was observed in both the ^19^F (*δ* −147.5 ppm, quartet, *J* = 8.6 Hz, Additional file 8) and ^11^B NMR spectra (*δ* 0.2 ppm, quartet, *J* = 8.5 Hz, Additional file 9) contributing 20 % to the total integrated signal. Its identity was assigned as a species containing one boron and three equivalent fluorine atoms (e.g. BF_3_OH^−^) owing to the quartet splitting pattern observed in the ^11^B spectrum, its similarity to the ^11^B spectrum of BF_3_·OEt_2_ in water (Additional file 10) and line broadening through quadrupolar relaxation due to loss of tetrahedral symmetry. This change in speciation was not observed without addition of acid. It was also accompanied by the appearance of an additional peak in the ^19^F spectrum corresponding to the release of fluoride and etching of the glass tube at acidic pH (SiF_6_^2−^*δ* −131 ppm, broad singlet, 13 % total integrated signal, Additional file 11). This interpretation is supported by the fact that this peak is also evident in the ^19^F spectrum of NaF in HCl (Additional file 12). After passing the acidified solution over an Ag^+^ cation exchange and an alumina column in tandem (to raise the pH of the solution by removing HCl and to remove fluoride, respectively), the suspected BF_3_OH^−^ species was no longer observed. Instead, the ^19^F spectrum showed only BF_4_^−^ (Additional file 7), while the ^11^B spectrum (Additional file 13) showed both BF_4_^−^ and an additional peak corresponding to boric acid B(OH)_3_, as confirmed by comparison to a standard solution of B(OH)_3_ (Additional file 14). As this transformation occurred upon passing over the alumina cartridge, and not the Ag^+^ cartridge, it was concluded that the alumina cartridge was catalysing this process.

### Radiosynthesis of [^18^F]BF_4_^−^ from BF_3_·OEt

To evaluate production of [^18^F]BF_4_^−^ from BF_3_·OEt (addition reaction) rather than from NaBF_4_ (isotopic substitution reaction), we initially investigated the use of several [^18^F]fluoride sources under anhydrous conditions. No labelled product was observed using [^18^F]KF/K[2.2.2]/K_2_CO_3_, but both [^18^F]NaF/15C5 and [^18^F]TBAF were found to give the desired product with high crude RCY (Additional file 2). To limit the amount of H_2_O present, [^18^F]NaF/15C5 was selected as the fluoride source to avoid the hygroscopic tetra n-butylammonium (TBA) salts. Precursor amount, temperature and time were then optimised further leading to a RCY of 86 % for the reaction step, as measured by TLC. Following alumina and QMA cartridge purification, this translated into an isolated decay-corrected RCY of 18.9 % (*n* = 2, decay-corrected to [^18^F]F^−^ trapping on QMA) and a synthesis time of 59 min from initial fluoride trapping. Most product loss occurred during the trapping of [^18^F]BF_4_^−^ on the QMA cartridge during purification, where ~50 % of the product failed to trap and was lost. Without the azeotropic drying step (i.e. in the presence of 0.9 % NaCl (0.4 mL)), no product was observed. Translation of the protocol to the GE FASTlab™ allowed production of [^18^F]BF_4_^−^ with radiochemical purity >95 %, decay-corrected RCY of 13.2 ± 5.9 % (*n* = 5) and synthesis time of 39 min from delivery of ^18^F^−^, in 0.9 % NaCl (final volume ~0.6 mL). The 15C5 in this product solution was 15–30 μg/mL (9–18 μg total, by ^1^H NMR, *n* = 3), and the pH was 7. The specific activity of the final product was 5.7 ± 3.5 GBq/μmol (*n* = 5) at the end of synthesis based on a starting radioactivity of ~1.5 GBq.

### In vivo imaging and biodistribution

**Fig. 2:**
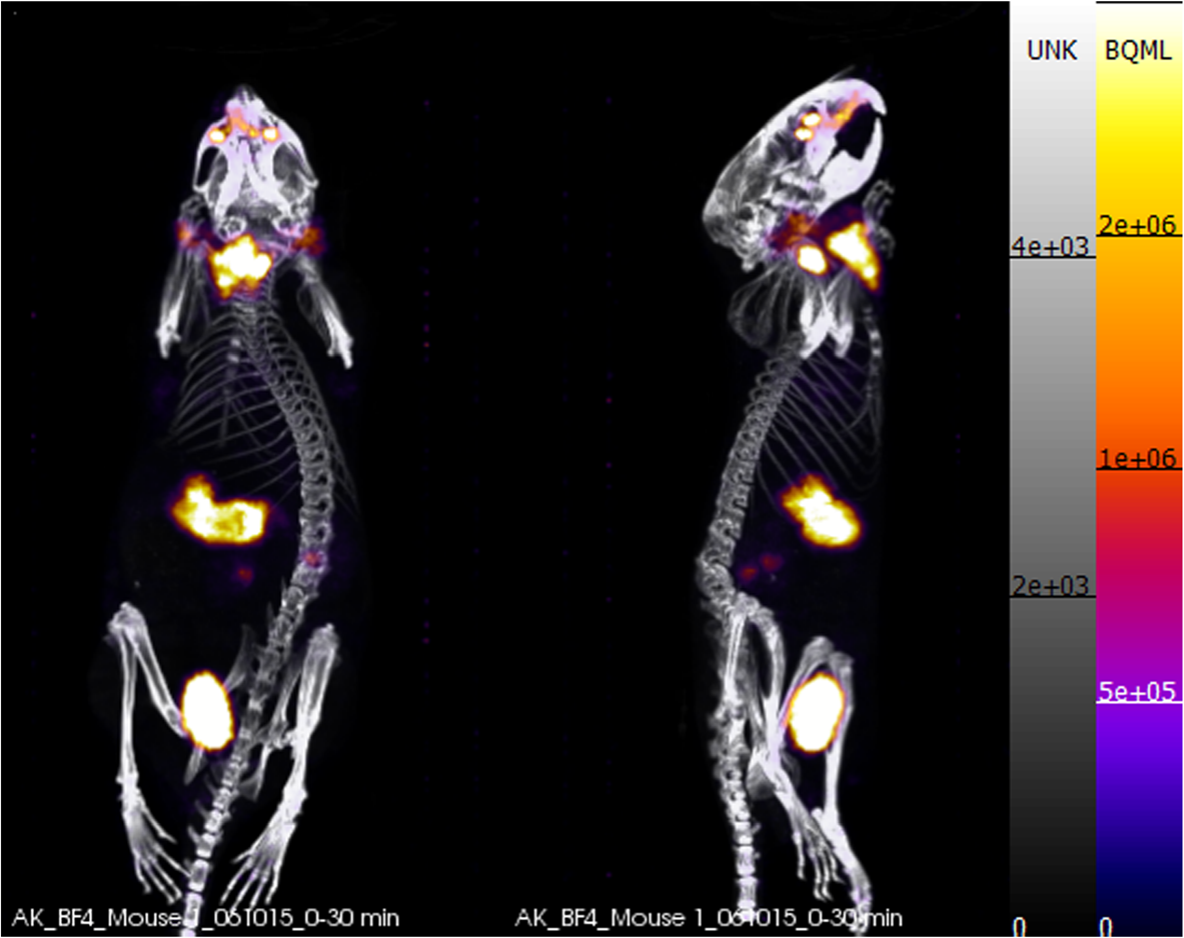
Side and anterior PET/CT maximum intensity projections of a normal BALB/c mouse 25–30 min post-injection of [^18^F]BF_4_^−^ (2.5 MBq, SA = 5 GBq/μmol) showing uptake in the thyroid, stomach, salivary gland and olfactory mucosa

**Fig. 3:**
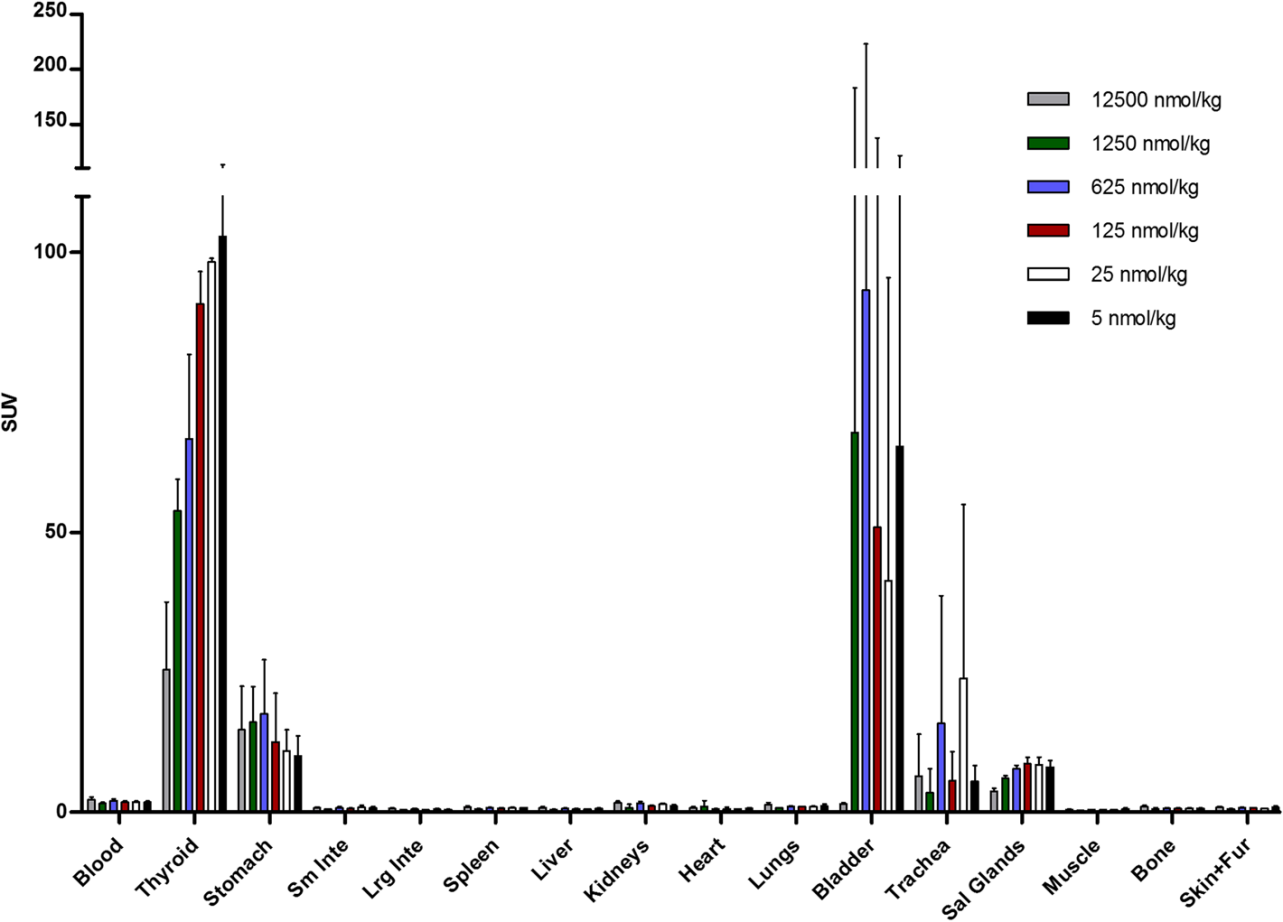
Ex vivo biodistribution data for [^18^F]BF_4_^−^ in BALB/c mice 45 min post-injection at varying doses of BF_4_^−^ (*n* = 3 for each dose) showing data for all tissues. Uptake is shown as a standardised uptake value (*SUV*). *Error bars* represent 1 SD. Of note is the trend of increasing/plateauing thyroid and salivary gland uptake as BF_4_^−^ dose (nmol/kg) decreases

**Fig. 4:**
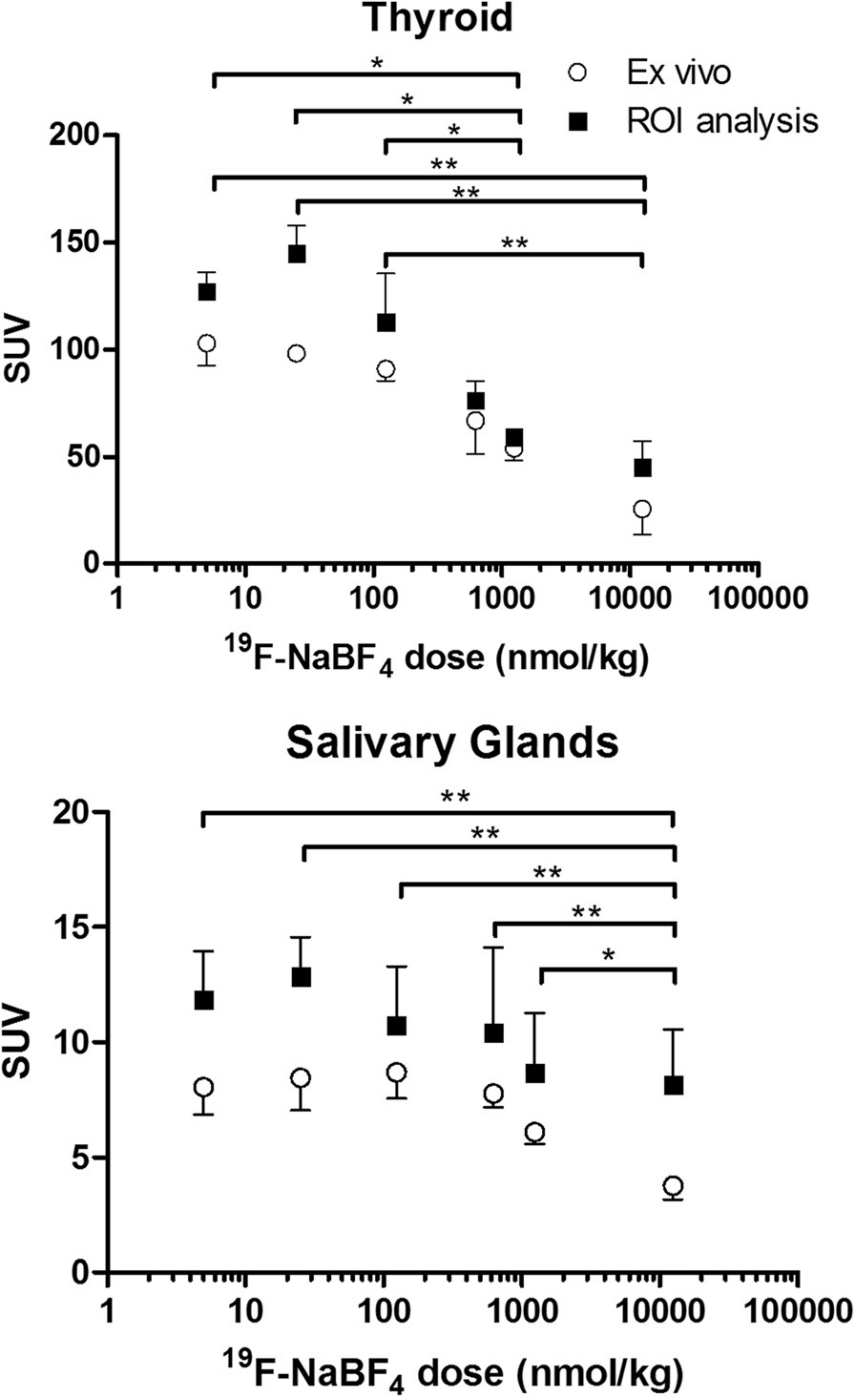
SUV for the thyroid (*upper*) and salivary glands (*lower*) in BALB/c mice estimated by ex vivo biodistribution (*open circles*) and PET ROI analysis (*filled squares*) at varying doses of ^18/19^F-BF_4_^−^ (*n* = 3 for each dose). *Error bars* represent 1 SD. *Significance bars* relate to the ex vivo data: *p < 0.05; **p < 0.01

As a basis for determining the effects of specific activity, [^18^F]BF_4_^−^ produced by the high-specific-activity method described in the “Methods” section was used and diluted with carrier ^19^F-BF_4_^−^ to produce samples of varying specific activity. As expected, significant uptake of the radiotracer in the thyroid, salivary glands and stomach was observed both by PET/CT (30 min post-injection, Fig. 2) and by ex vivo tissue counting (45 min post-injection, Fig. 3, shown as %ID/g in Additional files 4 and 5), with tracer excretion proceeding via the renal route. Uptake in the olfactory mucosa was visible in the PET images but was not analysed, and the tissue was not removed for ex vivo examination. Some trachea uptake was also observed in the ex vivo data, although this was not apparent in any of the images and hence may be attributable to incomplete removal of thyroid tissue from the trachea sample. SUV calculations for both the ex vivo biodistribution and the PET ROI analysis (Fig. 4, shown as %ID/g in Additional files 4 and 5) displayed a clear trend of decreasing uptake with increasing total BF_4_^−^ dose in both thyroid and salivary glands. Thyroid SUV reached a plateau at BF_4_^−^ doses below 125 nmol/kg but was significantly reduced at higher doses: for each of the three highest dose groups, it was significantly lower than for each of the two lowest dose groups (*p* < 0.05), with an ID_50_ of approximately 1000 nmol/kg. Similarly, salivary gland SUV reached a plateau at BF_4_^−^ doses below 125 nmol/kg but was significantly reduced at higher doses; at the highest dose, it was significantly lower than at the next highest dose (*p* < 0.05) and each of the four lowest doses (*p* < 0.01), with an ID_50_ of approximately 1000 nmol/kg. No discernible trend was apparent in the stomach, where uptake was highly variable within the groups. Statistical evaluation of inter-group differences by unpaired *t* test revealed no significant differences in any of the stomach data (Additional file 15).

## Discussion

The IC_50_ of BF_4_^−^ determined here (4.7 μM) and previously (1.6 μM [17]), as an inhibitor of hNIS in vitro suggests that the quantity of ^19^F-BF_4_^−^ in the radiopharmaceutical prepared by the published method [15] and administered in a dose sufficient for high-quality PET imaging could be in the range capable of adversely affecting radiotracer uptake via NIS. To avoid this risk, instantaneous in vivo extracellular BF_4_^−^ concentration should be kept below 0.1 μM. At the SA previously reported (1 GBq/μmol [15]), a typical human injection of (for example) 300 MBq [^18^F]BF_4_^−^ would contain 26 μg ^18/19^F-BF_4_^−^ (300 nmol) which upon initial in vivo dilution to an extracellular fluid volume of 14 L [21] would give a concentration of 21 nM before excretion takes effect. This concentration is well below that which might be expected to cause NIS inhibition. Predicted (based on the estimation described above) in vivo concentrations of BF_4_^−^ resulting from a 2.5-MBq injection of [^18^F]BF_4_^−^ into a BALB/c mouse (~20 g in weight, assuming an extracellular fluid volume of ~5 mL [22]) are illustrated on the IC_50_ curve depicted in Fig. 1. These estimates suggest that for a mouse injected with 2.5 MBq at the previously reported specific activity of 1 GBq/μg, the concentration would be 0.5 μM (corresponding to group (c) in Fig. 1). This is potentially sufficient to reduce the target uptake of the tracer and warrants experimental investigation of potential inhibition and development of a radiosynthesis that affords higher specific activity.

**Fig. 5:**
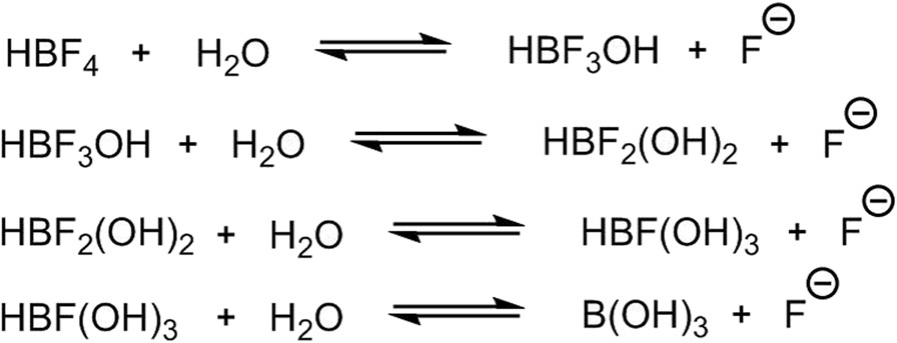
Sequential hydrolysis of BF_4_^−^ to B(OH)_3_

Our experience with the previously described isotopic exchange labelling method showed that the efficiency of the method was limited not only by the statistical need for a large excess of BF_4_^−^ (to achieve high yield in the isotopic exchange) but also by the alumina column used to remove residual [^18^F]fluoride. Despite the high incorporation of [^18^F]fluoride into [^18^F]BF_4_^−^ during the labelling reaction, a large proportion of the [^18^F]BF_4_^−^ was lost during this purification step, causing a substantial loss of yield. Further investigation of the species present at each stage of the labelling and purification by NMR led to the following conclusions:

1. BF_4_^−^ in aqueous solution at neutral pH exists as a single species with no appreciable B-F dissociation.
2. In strong acid, however, a minor but detectable proportion dissociates/hydrolyses to form a species containing a BF_3_ moiety, but no detectable further hydrolysis occurs to give species containing BF_2_ or BF moieties, nor further hydrolysis to borate during the time of the experiment. This is consistent with the observations of Wamser [23] that, under acidic conditions, BF_4_^−^ exists at equilibrium with BF_3_OH^−^ and F^−^.
3. Passage of the acidified solution through an alumina column traps fluoride, shifting the equilibrium (and probably catalysing the hydrolysis depicted in Fig. 5) to cause further hydrolysis of BF_4_^−^ ultimately to produce fluoride (which is retained on the column) and borate (which passes through the column and is detected by ^11^B NMR). This process has a major deleterious effect on the radiochemical yield but does not diminish the SA. The absence of BF_2_ and BF species in this instance indicates that these intermediates are unstable and their hydrolysis to borate is too rapid to be observed.

These equilibria are dependent on water, implying that aqueous acidic media for the labelling reaction cannot provide an efficient, high-specific-activity labelling reaction. Hence, there may be an advantage in developing a radiolabelling method that used only non-aqueous solvents until the final aqueous reconstitution stage.

**Fig. 6:**
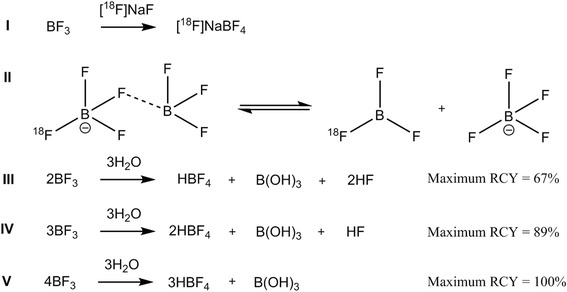
Theoretical chemical processes occurring in labelling conditions during production of [^18^F]BF_4_^−^ from BF_3_

Figure 6 lists a set of reaction equations representing possible scenarios to be considered in the reaction of BF_3_ with [^18^F]fluoride in the presence of traces of water. In the best case (equation I), simple addition of [^18^F]fluoride (or rather, substitution of diethyl ether) to BF_3_ would lead to [^18^F]BF_4_^−^, which in theory is separable from unreacted BF_3_. In this case, the SA would be limited only by the SA of the starting ^18^F^−^ (theoretical maximum 63,418 GBq/μmol [24]). In practice, due to systemic contamination with ^19^F^−^ from reagents and materials involved in production, a more typical SA is >5500 GBq/μmol [25]. This experimentally achievable SA is still >1000-fold higher than that obtained for [^18^F]BF_4_^−^; therefore, equation I alone is not sufficient to describe the labelling reaction. If fluoride exchange between BF_3_ and BF_4_^−^ (equation II) occurs to an appreciable extent under the reaction conditions, the yield would be diminished as well as the SA. Because of the very large excess of BF_3_ over the [^18^F]BF_4_^−^ product (which is limited by the trace amount of [^18^F]F^−^ added), the equilibrium represented by equation II would lead to very low yield; hence, the observed yield in excess of 80 % shows that equation II does not contribute significantly under these conditions. It is unrealistic to assume that water is completely absent even after azeotropic drying; therefore, equations III–V must also be considered. Both the SA and the yields observed are higher than achievable via equation III. Equation V would result in the lowest SA because all the ^19^F present in the precursor BF_3_·OEt_2_ would become incorporated into the final labelled BF_4_^−^ product which would contain 75 % of the starting boron. Using the amount of BF_3_·Et_2_O described in our method (0.115 mg) and 1.5 GBq of [^18^F]F^−^, the resulting SA would be 2.5 GBq/μmol, if the maximum possible yield of 100 % is achieved. As the observed SA is significantly higher than this, we can conclude that the reaction outlined in equation V is not a good fit to the data. Of the hydrolysis processes described in equations III to V, only equation IV is consistent with the observed yield and SA; it is likely that the other reactions contribute to a minor degree. The hydrolysis represented by equation IV has a minor deleterious effect on yield but a severely deleterious effect on SA; therefore, the primary requirement to achieve high specific activity is to eliminate water as far as possible, to maximise the contribution of equation I and minimise that of equation IV. If it is accepted that total exclusion of water is unrealistic and so some hydrolysis of BF_3_ [26] is inevitable, it then becomes important also to minimise the amount of BF_3_·Et_2_O used in the reaction and to avoid acidic pH (which catalyses hydrolysis).

The optimised labelling conditions reflect these requirements, by removing water and reducing the amount of BF_3_·Et_2_O to a minimum consistent with acceptable radiochemical yield and avoiding use of acid which catalyses hydrolysis. Labelling was entirely unsuccessful under typical K[2.2.2]/K_2_CO_3_ labelling conditions owing to the basic conditions and therefore [^18^F]NaF was selected as the fluoride source to maintain neutral pH and avoid H_2_O from being carried into the reaction mixture by TBA salts. A phase transfer catalyst, 15C5, was used to promote the nucleophilic reactivity of the fluoride but may also be capable of complexing BF_3_ [27]. The final reaction conditions produced the desired compound in a RCY and synthesis time suitable for clinical and preclinical use. Because of hydrolysis of BF_3_·Et_2_O, the SA reported here (5.7 GBq/μmol from a starting activity of 1.5 GBq) does not approach that theoretically attainable via equation I but is a marked improvement on that attainable by the previously published method (1 GBq/μmol from a starting activity of 10 GBq). While increasing the starting radioactivity of [^18^F]fluoride would not improve the SA in the absence of hydrolysis (equation I), under realistic reaction conditions, the SA should increase further with increasing starting radioactivity. This method was therefore adopted to produce [^18^F]BF_4_^−^ for in vivo investigation of the effect of varying specific activity.

Both the ex vivo biodistribution data and ROI analysis of PET/CT images indicate a trend of increasing uptake in thyroid and salivary gland tissue with increasing SA (Fig. 4), with an ID_50_ of around 1000 nmol/kg and thyroid and salivary gland SUV plateauing above 100 nmol/kg. The stomach, by contrast, did not display any discernible relationship between SUV and BF_4_^−^ dose, perhaps because of high variance and interference from alternative anion-transporting proteins and alternative weakly competing substrates of NIS in the gastric mucosa. The in vivo data (Fig. 4) are thus broadly consistent with projections from the in vitro data (Fig. 1), which suggest that to achieve maximum target-to-background ratio, the instantaneous extracellular BF_4_^−^ concentration in vivo should not exceed 0.1 μM. To achieve this in mice with an injected activity of 2.5 MBq would require a SA of at least 5 GBq/μmol assuming an extracellular volume of 5 mL. This SA is readily achievable with the addition reaction described here, but not with the previously published isotopic substitution method [15].

## Conclusions

To avoid saturation of [^18^F]BF_4_^−^ uptake by NIS-expressing tissues, the administered mass of BF_4_^−^ should be kept below 100 nmol/kg (~11 μg/kg NaBF_4_) in mice. This is achievable in humans even with the low-SA synthesis reported previously [15], but in mice, it demands a higher SA. This difference between mouse and human imaging has been noted previously [18]. Therefore, we have developed a fully automated radiosynthesis which optimises the SA that can realistically be achieved. While the SA reported herein (5.7 GBq/μmol from a starting radioactivity of only 1.5 GBq) is sufficient to avoid compromising uptake, it could be improved further by increasing the starting radioactivity. The improved SA is particularly important in view of the growing use of hNIS PET as a preclinical research tool for reporter gene imaging.

### Statement of ethics approval

Animal experiments were conducted in accordance with Medical Research Charities’ and UK Research Councils’ guidance on Responsibility in the Use of Animals in Bioscience Research, under a UK Home Office licence and approved by a local KCL animal ethics committee.

## Additional files

Additional file 1: Radioisotopes used for theranostic applications and reporter gene imaging with NIS. (PDF 73.9 KB). Additional file 2: Reaction conditions tested, and RCY obtained under each set of conditions, during optimisation of radiochemistry. (PDF 20.8 KB). Additional file 3: GE FASTlab™ cassette layout for [^18^ F]BF_4_^−^ synthesis. (PDF 180 KB). Additional file 4: %ID/g for the thyroid (upper), salivary glands (centre) and stomach (lower) in BALB/c mice estimated by ex vivo biodistribution (open circles) and PET ROI analysis (filled squares) at varying doses of ^18/19^F-BF_4_^−^. (PDF 15.7 KB). Additional file 5: Ex vivo biodistribution data for [^18^F]BF_4_^−^ in BALB/c mice 45 min post-injection at varying doses of BF_4_^−^. (PDF 18.1 KB). Additional file 6: Expanded view of ^11^B NMR spectrum of NaBF_4_ in neutral H_2_O/D_2_O. (PDF 11.6 KB). Additional file 7: Expanded view of ^19^F NMR spectrum of NaBF_4_ in neutral H_2_O/D_2_O. (PDF 75.4 KB). Additional file 8: Changes in the ^19^F NMR spectra throughout the isotopic exchange labelling process. (PDF 107 KB). Additional file 9: Expanded view of ^11^B NMR spectrum of NaBF_4_ after being heated in HCl. (PDF 77.7 KB). Additional file 10:
^11^B NMR of BF_3_ ·OEt^2^ and NaBF_4_ in H_2_O. (PDF 25.0 KB). Additional file 11:
^19^F NMR spectrum of NaBF4 after being heated in HCl. (PDF 90.0 KB). Additional file 12:
^19^F NMR spectra of NaF at neutral and acidic pH in quartz tubes. (PDF 96.2 KB). Additional file 13: Changes in the ^11^B NMR spectra of NaBF_4_ throughout the isotopic exchange labelling process. (PDF 65.0 KB). Additional file 14: Expanded view of ^11^B NMR spectrum of boric acid. (PDF 12.5 KB). Additional file 15: SUV for the stomach in BALB/c mice estimated by ex vivo biodistribution and PET ROI analysis at varying doses of BF_4_^−^. (PDF 9.33 KB).

## Abbreviations

15C5: 15-crown-5
BF_3_·OEt_2_: boron trifluoride diethyl etherate
CT: computed tomography
HBSS: Hanks’ balanced salt solution
hNIS: human sodium/iodide symporter
IC: ion chromatography
IC_50_: half-maximal inhibitory concentration
ID_50_: half-maximal inhibitory dose
K[2.2.2]: Kryptofix 2.2.2
MeCN: acetonitrile
NIS: sodium/iodide symporter
NMR: nuclear magnetic resonance
PET: positron emission tomography
QMA: quaternary methyl ammonium
RCY: radiochemical yield
ROI: region of interest
SA: specific activity
SPECT: single photon emission computed tomography
SUV: standardised uptake value
TBA: tetra n-butylammonium
TBAF: tetra n-butylammonium fluoride
TBR: target-to-background ratio
TLC: thin-layer chromatography
