# Supplementary material for: [18F]tetrafluoroborate as a PET tracer for the sodium/iodide symporter: the importance of specific activity
Source: EJNMMI Res. 2016 Apr 22;6:34. doi: 10.1186/s13550-016-0188-5 (PMC4840125; doi:10.1186/s13550-016-0188-5)
Supplement: Additional file 1: — Radioisotopes used for theranostic applications and reporter gene imaging with NIS. (PDF 73.9 KB). [file 13550_2016_188_MOESM1_ESM.pdf]

Radioisotope	Chemical Form	Half-life	Emission	Application
**^{123}I**	I^-	13.2 h	γ	SPECT imaging
**^{124}I**	I^-	4.2 d	β^+/γ	PET imaging
**^{131}I**	I^-	8.0 d	β^-/γ	SPECT imaging /radionuclide therapy
**^{211}At**	At^-	7.2 h	α	Radionuclide therapy
**^{99m}Tc**	TcO_4^-	6.0 h	γ	SPECT imaging
**^{186}Re**	ReO_4^-	90.6 h	β^-/γ	Radionuclide therapy
**^{188}Re**	ReO_4^-	17.0 h	β^-/γ	Radionuclide therapy
**^{18}F**	BF_4^-	109.8 min	β^+	PET imaging

Radioisotopes used for theranostic applications and reporter gene imaging with NIS
