# Supplementary material for: [18F]tetrafluoroborate as a PET tracer for the sodium/iodide symporter: the importance of specific activity
Source: EJNMMI Res. 2016 Apr 22;6:34. doi: 10.1186/s13550-016-0188-5 (PMC4840125; doi:10.1186/s13550-016-0188-5)
Supplement: Additional file 2: — Reaction conditions tested, and RCY obtained under each set of conditions, during optimisation of radiochemistry. (PDF 20.8 KB). [file 13550_2016_188_MOESM2_ESM.pdf]

$^{18}F^-$ source	QMA Eluent	Phase transfer catalyst (mg)	Reaction time (min)	Temp. (°C)	Precursor Amount (µL)	Radiochemical yield* (%)
KF	K_2CO_3	K[2.2.2] (7.6)	10	80	0.1	0
TBAF	$TBANO_3$	-	10	80	0.1	84
NaF	NaCl	15C5 (23)	1	90	10	58
NaF	NaCl	15C5 (23)	1	90	1	16
NaF	NaCl	15C5 (23)	1	90	0.1	19
NaF	NaCl	15C5 (23)	1	90	0.01	9
NaF	NaCl	15C5 (23)	1	90	0.001	0
NaF	NaCl	15C5 (23)	1	90	0.0001	0
NaF	NaCl	15C5 (23)	10	80	0.1	86
NaF	NaCl	15C5 (23)	10	70	0.1	83
NaF	NaCl	15C5 (23)	10	55	0.1	52
NaF	NaCl	15C5 (23)	10	40	0.1	20
NaF	NaCl	15C5 (23)	5	80	0.1	71
NaF	NaCl	15C5 (23)	10	80	0.1	70
NaF	NaCl	15C5 (23)	15	80	0.1	79
NaF	NaCl	15C5 (23)	20	80	0.1	90
NaF	NaCl	15C5 (23)	30	80	0.1	68
NaF	NaCl	15C5 (46)	5	80	0.1	75
NaF	NaCl	-	5	80	0.1	50

Reaction conditions tested, and RCY obtained under each set of conditions, during optimisation of radiochemistry *non-isolated, measured by TLC of the crude product
