# Supplementary material for: [18F]tetrafluoroborate as a PET tracer for the sodium/iodide symporter: the importance of specific activity
Source: EJNMMI Res. 2016 Apr 22;6:34. doi: 10.1186/s13550-016-0188-5 (PMC4840125; doi:10.1186/s13550-016-0188-5)
Supplement: Additional file 3: — GE FASTlab™ cassette layout for [18 F]BF4 − synthesis. (PDF 180 KB). [file 13550_2016_188_MOESM3_ESM.pdf]

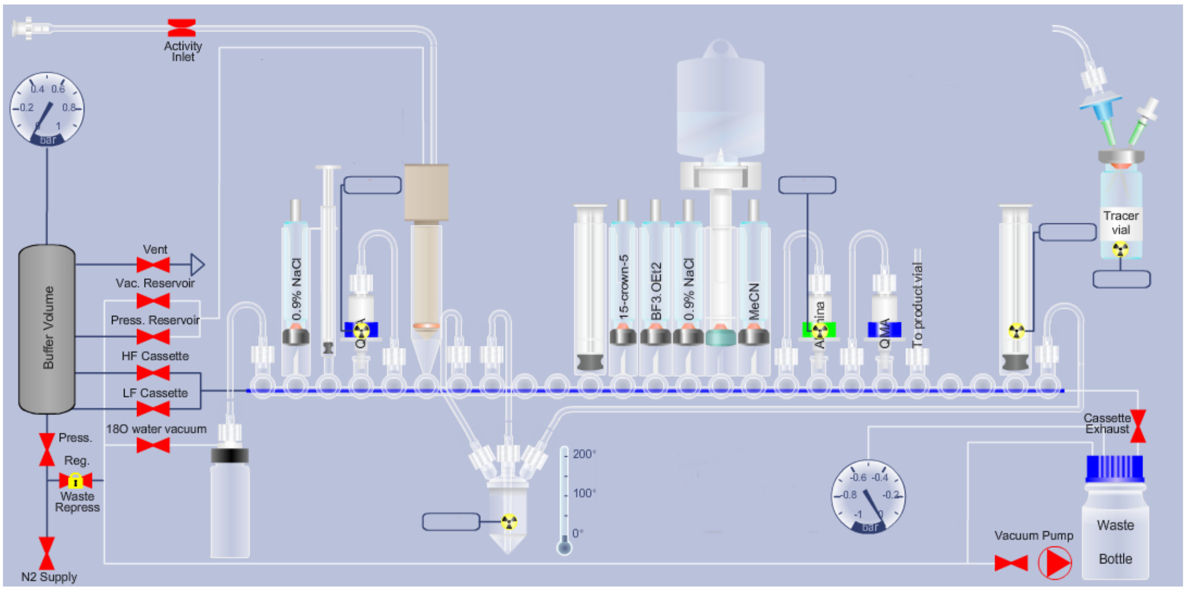

GE FASTlab™ cassette layout for $[^{18}F]BF_4^-$ synthesis. SPE cartridges are conditioned as in the main manuscript. Reagents are present in cassette positions 2 (0.9% NaCl, 750 µL), 12 (15-crown-5, 46 mg in MeCN (800 µL)), 13 ($BF_3.OEt_2$, 0.2 µL in MeCN (850 µL)), 14 (0.9% NaCl, 1 mL), 15 (H_2O (sterile purified water, GE Healthcare)) and 16 (MeCN, 1.5 mL).
