# Supplementary material for: [18F]tetrafluoroborate as a PET tracer for the sodium/iodide symporter: the importance of specific activity
Source: EJNMMI Res. 2016 Apr 22;6:34. doi: 10.1186/s13550-016-0188-5 (PMC4840125; doi:10.1186/s13550-016-0188-5)
Supplement: Additional file 4: — %ID/g for the thyroid (upper), salivary glands (centre) and stomach (lower) in BALB/c mice estimated by ex vivo biodistribution (open circles) and PET ROI analysis (filled squares) at varying doses of 18/19F-BF4 −. (PDF 15.7 KB). [file 13550_2016_188_MOESM4_ESM.pdf]

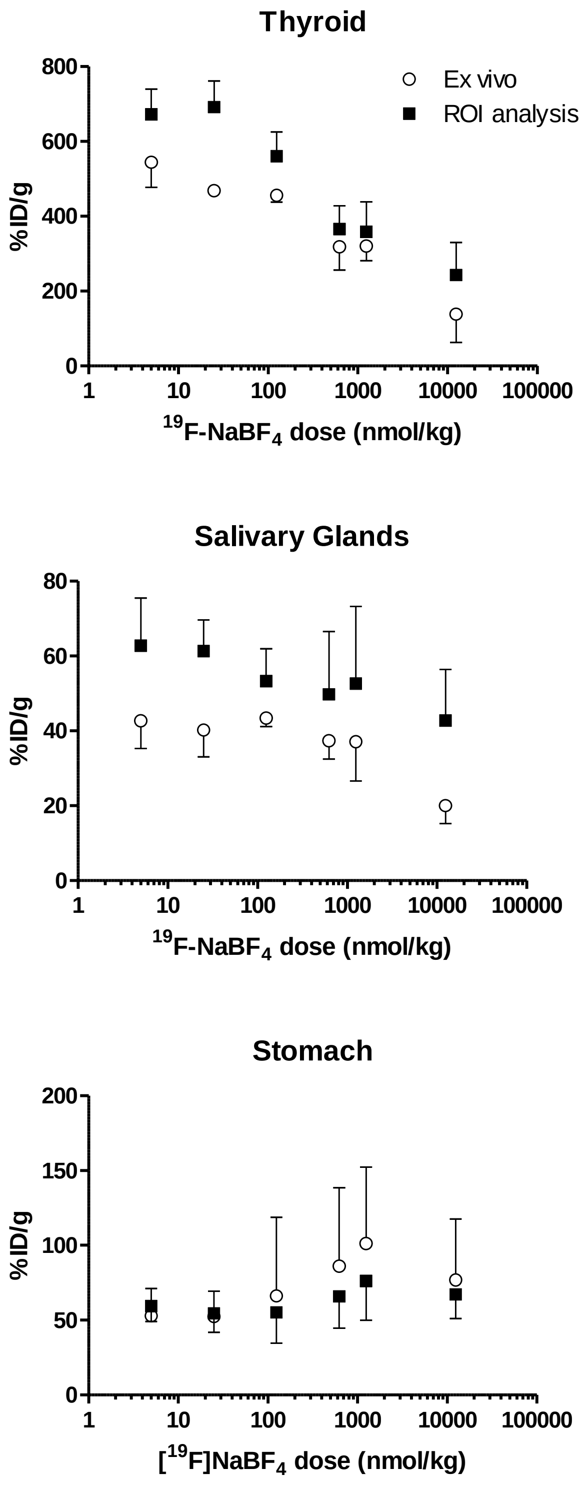


%ID/g for thyroid (upper), salivary glands (center) and stomach (lower) in Balb/c mice estimated by *ex vivo* biodistribution (open circles) and PET ROI analysis (filled squares) at varying doses of $^{18/19}F\text{-}BF_4^-$ (n = 3 for each dose). Error bars represent 1 SD.
