# Supplementary material for: [18F]tetrafluoroborate as a PET tracer for the sodium/iodide symporter: the importance of specific activity
Source: EJNMMI Res. 2016 Apr 22;6:34. doi: 10.1186/s13550-016-0188-5 (PMC4840125; doi:10.1186/s13550-016-0188-5)
Supplement: Additional file 5: — Ex vivo biodistribution data for [18F]BF4 − in BALB/c mice 45 min post-injection at varying doses of BF4 −. (PDF 18.1 KB). [file 13550_2016_188_MOESM5_ESM.pdf]

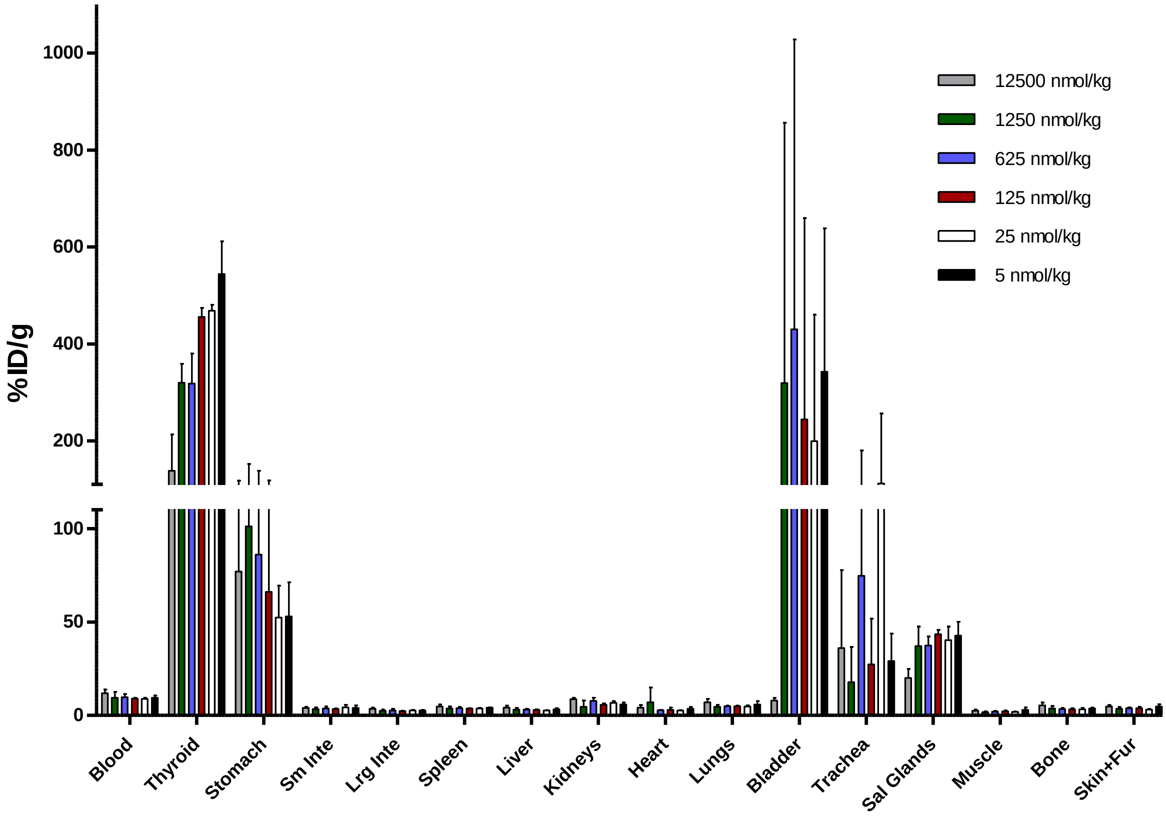

Ex vivo biodistribution data for $[^{18}F]BF_4^-$ in Balb/c mice 45 min post-injection at varying doses of BF_4^- (n = 3 for each dose) showing data for all tissues. Uptake is shown as injected dose per gram tissue mass (%ID/g). Error bars represent 1 SD. Of note is the trend of increasing/plateauing thyroid and salivary gland uptake as BF_4^- dose (nmol/kg) decreases.
