# Supplementary figures and images for: [18F]tetrafluoroborate as a PET tracer for the sodium/iodide symporter: the importance of specific activity
Source: EJNMMI Res. 2016 Apr 22;6:34. doi: 10.1186/s13550-016-0188-5 (PMC4840125; doi:10.1186/s13550-016-0188-5)

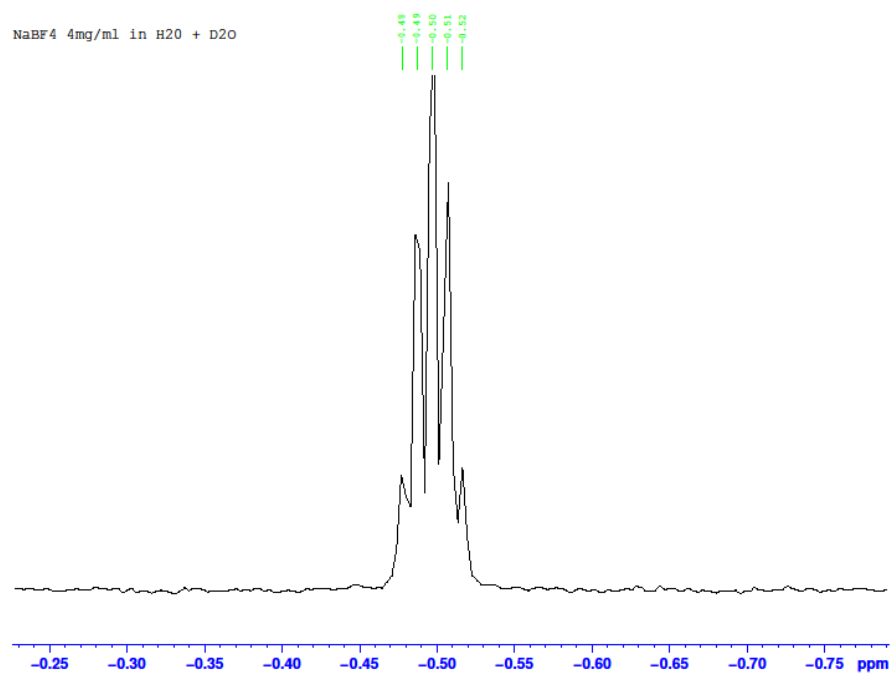

Expanded view of <sup>11</sup>B NMR spectrum of NaBF<sub>4</sub> (4 mg/mL) in neutral H<sub>2</sub>O/D<sub>2</sub>O

Supplement: Additional file 6: — Expanded view of 11B NMR spectrum of NaBF4 in neutral H2O/D2O. (PDF 11.6 KB). [file 13550_2016_188_MOESM6_ESM.pdf]

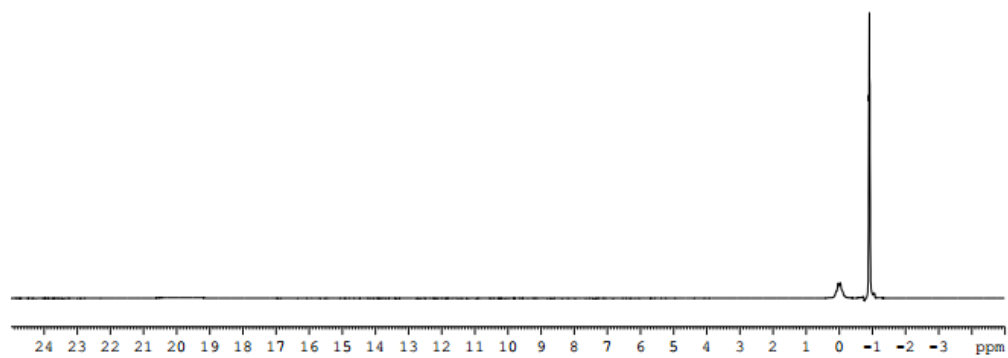

$^{11}\text{B}$  NMR of  $\text{BF}_3\text{OEt}_2$  ( $\delta$  0 ppm) and  $\text{NaBF}_4$  ( $\delta$  -1 ppm) in  $\text{H}_2\text{O}$

Supplement: Additional file 10: — 11B NMR of BF3 ·OEt2 and NaBF4 in H2O. (PDF 25.0 KB). [file 13550_2016_188_MOESM10_ESM.pdf]
