# Supplementary material for: [18F]tetrafluoroborate as a PET tracer for the sodium/iodide symporter: the importance of specific activity
Source: EJNMMI Res. 2016 Apr 22;6:34. doi: 10.1186/s13550-016-0188-5 (PMC4840125; doi:10.1186/s13550-016-0188-5)
Supplement: Additional file 7: — Expanded view of 19F NMR spectrum of NaBF4 in neutral H2O/D2O. (PDF 75.4 KB). [file 13550_2016_188_MOESM7_ESM.pdf]

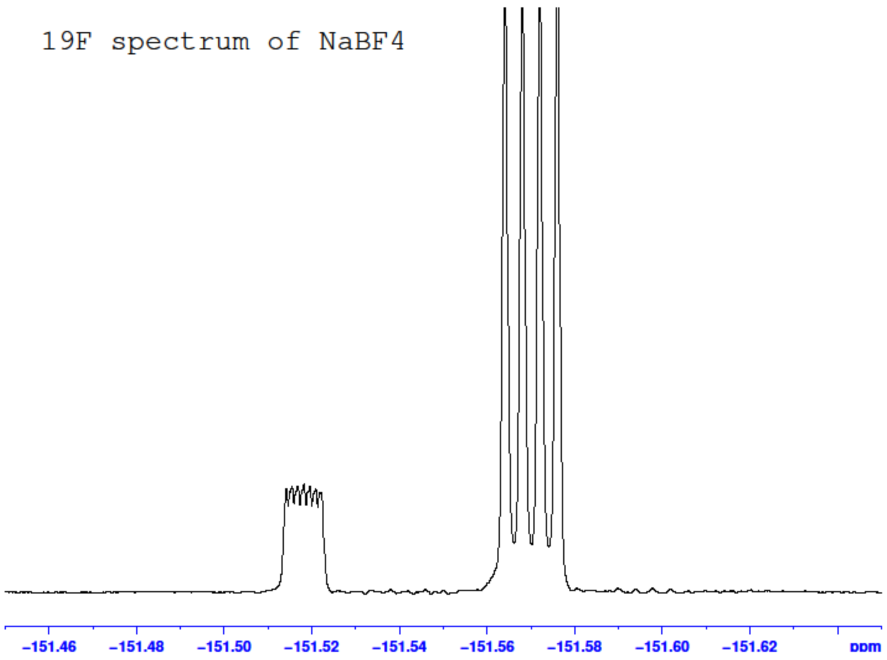

Expanded view of ^{19}F NMR spectrum of $NaBF_4$ (4 mg/mL) in neutral H_2O/D_2O. The two multiplets correspond to $^{10}BF_4^-$ (δ-151.52, septet) and $^{11}BF_4^-$ (δ-151.57, quartet) with the difference in chemical shift resulting from differing equilibrium bond lengths due to vibrational effects owing to the change in mass between ^{10}B and ^{11}B.
