# Supplementary material for: [18F]tetrafluoroborate as a PET tracer for the sodium/iodide symporter: the importance of specific activity
Source: EJNMMI Res. 2016 Apr 22;6:34. doi: 10.1186/s13550-016-0188-5 (PMC4840125; doi:10.1186/s13550-016-0188-5)
Supplement: Additional file 8: — Changes in the 19F NMR spectra throughout the isotopic exchange labelling process. (PDF 107 KB). [file 13550_2016_188_MOESM8_ESM.pdf]

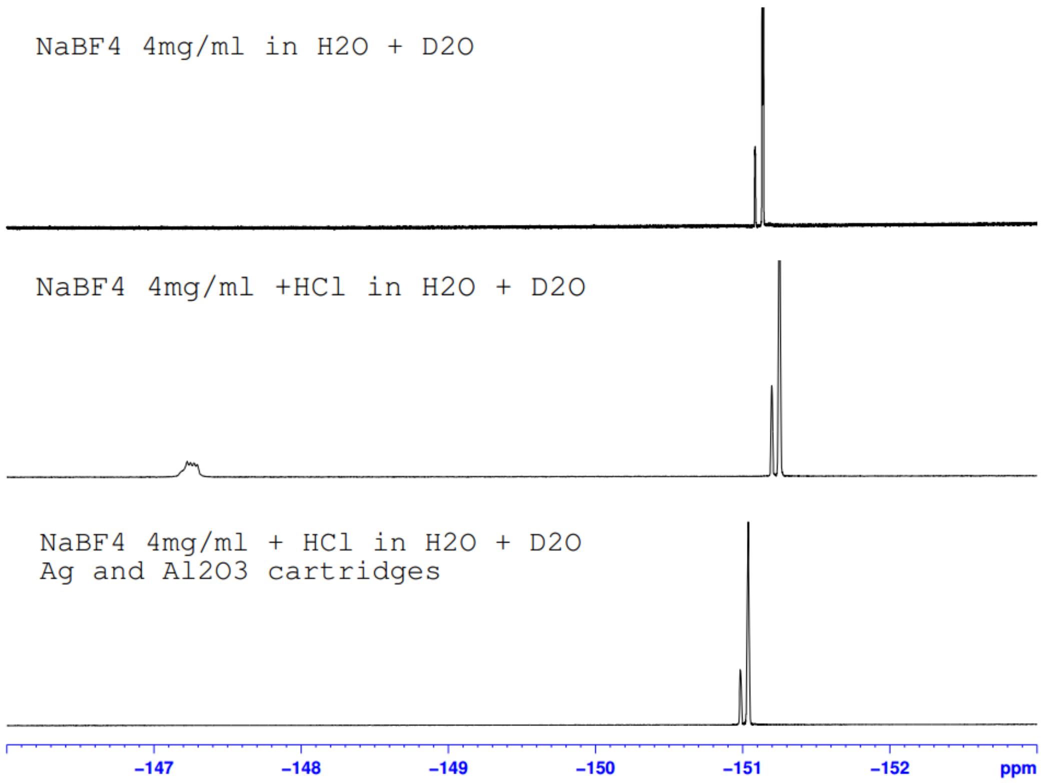

Changes in the ^{19}F NMR spectra throughout the isotopic exchange labelling process: in neutral solution, in acidified labelling solution, and after passing over both Ag and alumina cartridges (top to bottom). At neutral pH, F^- would be visible at δ -121 ppm as a singlet while at acidic pH (1 M HCl) would appear at δ -131 ppm as a broad singlet. Conditions given in Methods section of main manuscript.
