# Supplementary material for: [18F]tetrafluoroborate as a PET tracer for the sodium/iodide symporter: the importance of specific activity
Source: EJNMMI Res. 2016 Apr 22;6:34. doi: 10.1186/s13550-016-0188-5 (PMC4840125; doi:10.1186/s13550-016-0188-5)
Supplement: Additional file 9: — Expanded view of 11B NMR spectrum of NaBF4 after being heated in HCl. (PDF 77.7 KB). [file 13550_2016_188_MOESM9_ESM.pdf]

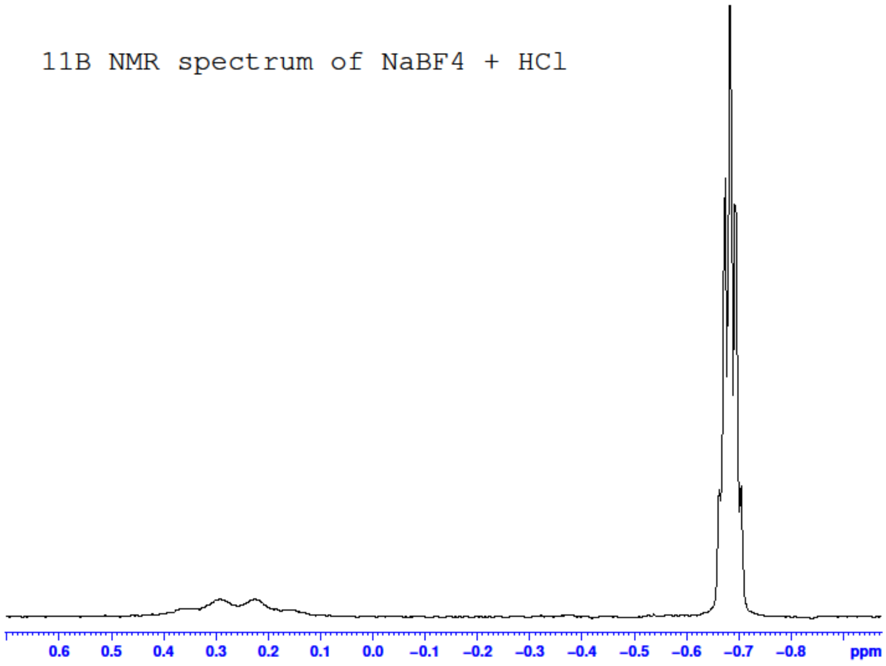

Expanded view of ^{11}B NMR spectrum of $NaBF_4$ (4 mg/mL) after being heated in HCl (1 M). Peaks correspond to BF_4^- (δ -0.7 ppm, quintet, J = 1.3 Hz) and suspected BF_3OH^- (δ -0.2 ppm, quartet, J = 8.5 Hz).
