# Supplementary material for: [18F]tetrafluoroborate as a PET tracer for the sodium/iodide symporter: the importance of specific activity
Source: EJNMMI Res. 2016 Apr 22;6:34. doi: 10.1186/s13550-016-0188-5 (PMC4840125; doi:10.1186/s13550-016-0188-5)
Supplement: Additional file 11: — 19F NMR spectrum of NaBF4 after being heated in HCl. (PDF 90.0 KB). [file 13550_2016_188_MOESM11_ESM.pdf]

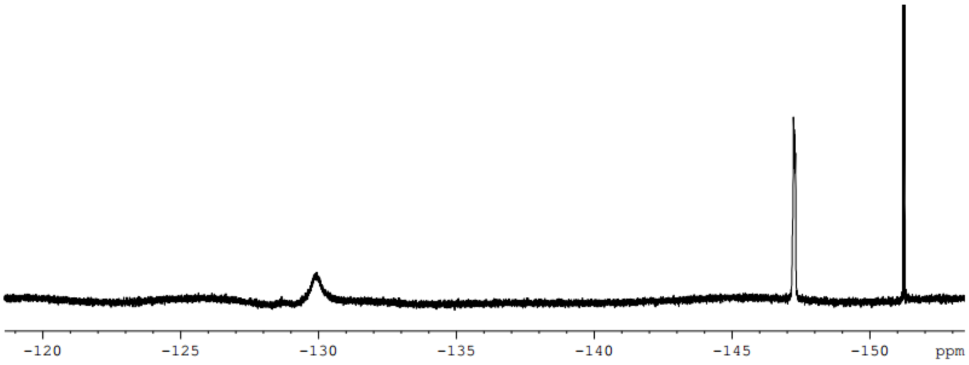

Zoomed out view of $NaBF_4$ (4 mg/mL) after being heated in HCl (1 M) showing SiF_6^{2-} resulting from glass etching following F^- release (δ -131 ppm, broad singlet), proposed BF_3OH^- (δ -147.5 ppm, quartet) and BF_4^- ($^{10}BF_4^-$ (δ-151.52, septet) and $^{11}BF_4^-$ (δ-151.57, quartet)).
