# Supplementary material for: [18F]tetrafluoroborate as a PET tracer for the sodium/iodide symporter: the importance of specific activity
Source: EJNMMI Res. 2016 Apr 22;6:34. doi: 10.1186/s13550-016-0188-5 (PMC4840125; doi:10.1186/s13550-016-0188-5)
Supplement: Additional file 12: — 19F NMR spectra of NaF at neutral and acidic pH in quartz tubes. (PDF 96.2 KB). [file 13550_2016_188_MOESM12_ESM.pdf]

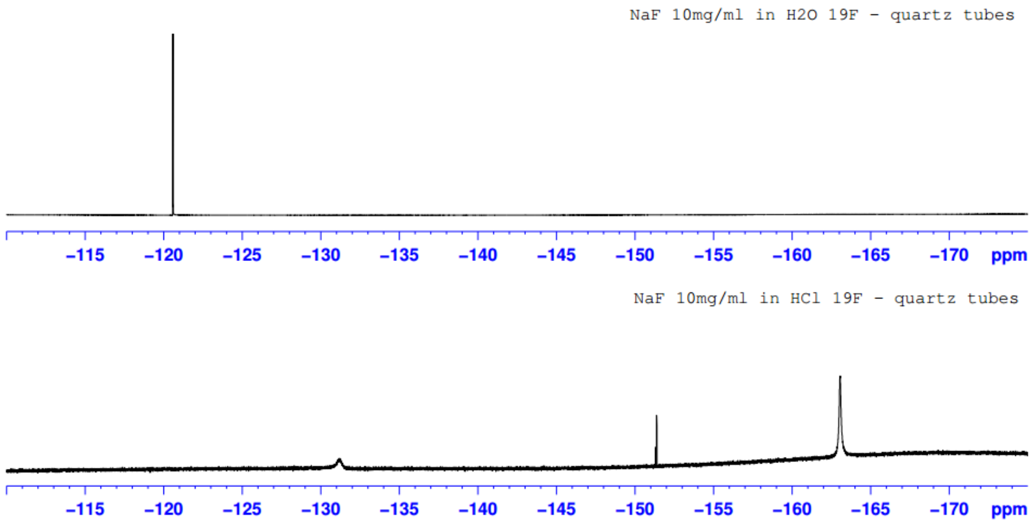

^{19}F NMR spectra of NaF (4 mg/mL) at neutral (H_2O) and acidic (1 M HCl) pH in quartz tubes to demonstrate the behaviour of fluoride at neutral and acidic pH, included as a reference. Shows the presence of only F^- (δ -121 ppm, singlet) at neutral pH while at acidic pH SiF_6^{2-} (δ -131 ppm, broad singlet), BF_4^- ($^{10}BF_4^-$ (δ-151.52, septet) and $^{11}BF_4^-$ (δ-151.57, quartet)) and HF/F^- H-bonded species (δ -163 ppm, broad singlet) are all present.
