# Supplementary material for: [18F]tetrafluoroborate as a PET tracer for the sodium/iodide symporter: the importance of specific activity
Source: EJNMMI Res. 2016 Apr 22;6:34. doi: 10.1186/s13550-016-0188-5 (PMC4840125; doi:10.1186/s13550-016-0188-5)
Supplement: Additional file 13: — Changes in the 11B NMR spectra of NaBF4 throughout the isotopic exchange labelling process. (PDF 65.0 KB). [file 13550_2016_188_MOESM13_ESM.pdf]

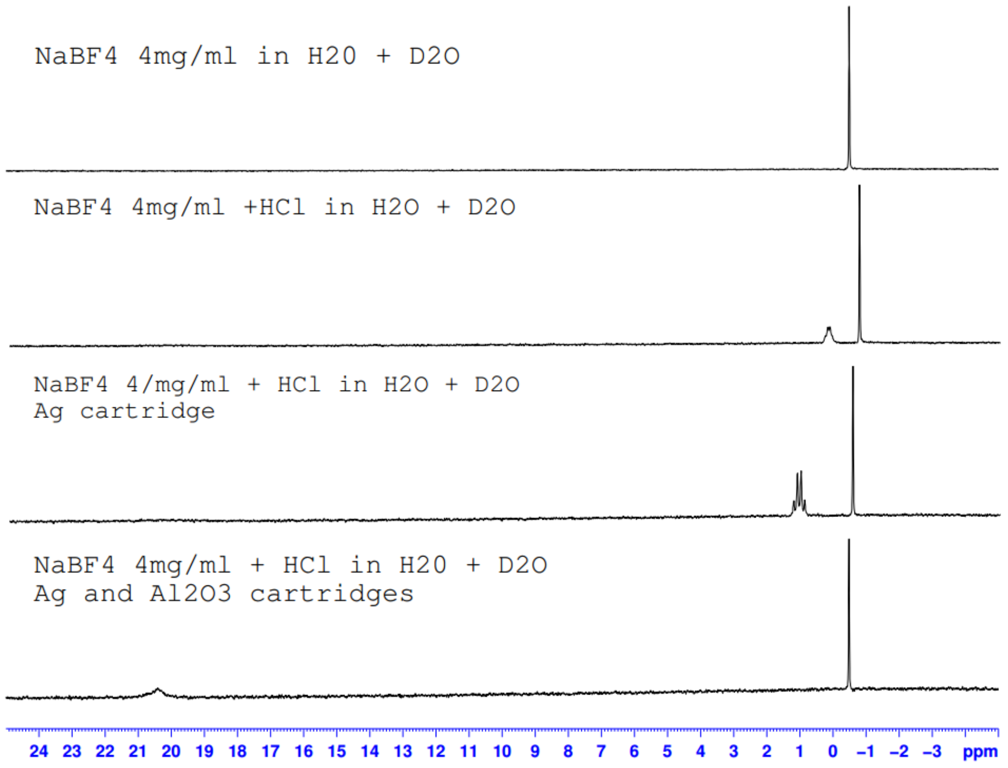

Changes in the ^{11}B NMR spectra of $NaBF_4$ throughout the isotopic exchange labelling process: in neutral solution, in acidified labelling solution, after passing over Ag cartridge and after passing over both Ag and alumina cartridges (top to bottom). Conditions given in Methods section of main manuscript.
