# Supplementary material for: [18F]tetrafluoroborate as a PET tracer for the sodium/iodide symporter: the importance of specific activity
Source: EJNMMI Res. 2016 Apr 22;6:34. doi: 10.1186/s13550-016-0188-5 (PMC4840125; doi:10.1186/s13550-016-0188-5)
Supplement: Additional file 14: — Expanded view of 11B NMR spectrum of boric acid. (PDF 12.5 KB). [file 13550_2016_188_MOESM14_ESM.pdf]

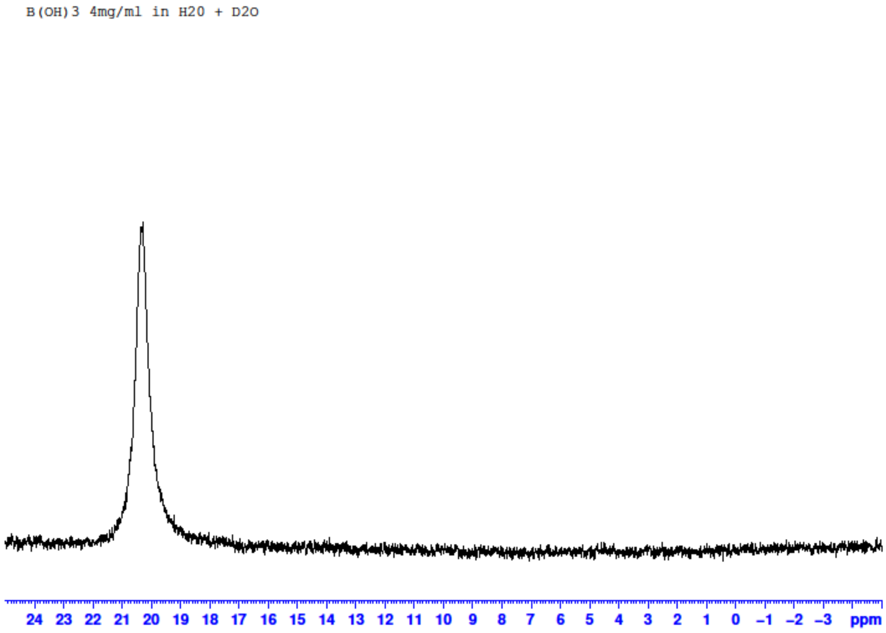

Expanded view of ^{11}B NMR spectrum of boric acid, $B(OH)_3$ (4 mg/mL) in neutral H_2O/D_2O
