# Supplementary material for: [18F]tetrafluoroborate as a PET tracer for the sodium/iodide symporter: the importance of specific activity
Source: EJNMMI Res. 2016 Apr 22;6:34. doi: 10.1186/s13550-016-0188-5 (PMC4840125; doi:10.1186/s13550-016-0188-5)
Supplement: Additional file 15: — SUV for the stomach in BALB/c mice estimated by ex vivo biodistribution and PET ROI analysis at varying doses of BF4 −. (PDF 9.33 KB). [file 13550_2016_188_MOESM15_ESM.pdf]

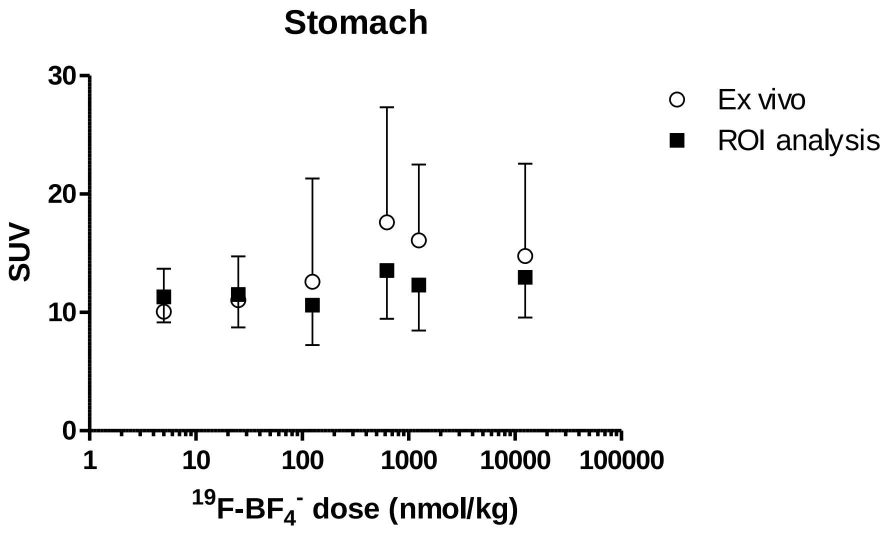

SUV for stomach in Balb/c mice estimated by *ex vivo* biodistribution (open circles) and PET ROI analysis (filled squares) at varying doses of BF_4^- (n = 3 for each dose). Error bars represent 1 SD.
